# Supplementary material for: Smooth Interpolating Curves with Local Control and Monotone Alternating Curvature
Source: Comput Graph Forum. 2022 Oct 6;41(5):25–38. doi: 10.1111/cgf.14600 (PMC9827861; doi:10.1111/cgf.14600)
Supplement: Supplementary file 1 — Supplement Material [file CGF-41-25-s001.zip › Local-Smooth-Interpolating-MonoCurvature/extern/clothoids/submodules/Utils/docs/api-cpp/program_listing_file_Utils_Utils.hxx.html]

Program Listing for File Utils.hxx — Utils v1.2

### Navigation

- index
- toc
- Utils »
- Program Listing for File Utils.hxx

# Program Listing for File Utils.hxx¶

↰ Return to documentation for file (`Utils/Utils.hxx`)

```
/*--------------------------------------------------------------------------*\
 |                                                                          |
 |  Copyright (C) 2017                                                      |
 |                                                                          |
 |         , __                 , __                                        |
 |        /|/  \               /|/  \                                       |
 |         | __/ _   ,_         | __/ _   ,_                                |
 |         |   \|/  /  |  |   | |   \|/  /  |  |   |                        |
 |         |(__/|__/   |_/ \_/|/|(__/|__/   |_/ \_/|/                       |
 |                           /|                   /|                        |
 |                           \|                   \|                        |
 |                                                                          |
 |      Enrico Bertolazzi                                                   |
 |      Dipartimento di Ingegneria Industriale                              |
 |      Universita` degli Studi di Trento                                   |
 |      email: enrico.bertolazzi@unitn.it                                   |
 |                                                                          |
\*--------------------------------------------------------------------------*/


#pragma once

#ifndef DOXYGEN_SHOULD_SKIP_THIS
#ifndef UTILS_dot_HXX
#define UTILS_dot_HXX
#endif

// select computer architecture
#if defined(__APPLE__) && defined(__MACH__)
  // osx architecture
  #define UTILS_OS_OSX 1
  #if defined(__i386__)
    #define UTILS_ARCH32 1
  #elif defined(__x86_64__)
    #define UTILS_ARCH64 1
  #endif
#elif defined(__unix__)
  // linux architecture
  #define UTILS_OS_LINUX 1
  #if defined(__i386__)
    #define UTILS_ARCH32 1
  #elif defined(__x86_64__)
    #define UTILS_ARCH64 1
  #endif
#elif defined(_WIN32) || defined(WIN32) || defined(_WIN64) || defined(WIN64)
  // windows architecture
  #define UTILS_OS_WINDOWS 1
  #if defined(_M_X64) || defined(_M_AMD64) || defined(_WIN64) || defined(WIN64)
    #define UTILS_ARCH64 1
  #else
    #define UTILS_ARCH32 1
  #endif
#else
  #error "unsupported OS!"
#endif

// check if compiler is C++11
#if (defined(_MSC_VER) &&  _MSC_VER >= 1800) || \
    (defined(__cplusplus) && __cplusplus > 199711L)
#else
  #error "Lapack Wrapper must be compiled using C++ >= C++11"
#endif

#define UTILS_PURE_VIRTUAL = 0
#ifndef UTILS_OS_WINDOWS
  #define UTILS_OVERRIDE  override
  #define UTILS_CONSTEXPR constexpr
#else
  #define UTILS_OVERRIDE
  #define UTILS_CONSTEXPR
#endif

#ifndef DOXYGEN_SHOULD_SKIP_THIS
#include "fmt/printf.h"
#include "fmt/chrono.h"
#include "fmt/ostream.h"
#endif

#include <string>
#include <iostream>
#include <iomanip>
#include <sstream>
#include <cstdlib>
#include <cmath>
#include <cstdint>
#include <stdexcept>

// disable mingw-std-threads for mingw on MATLAB
#if (defined(MINGW) || defined(__MINGW32__)) && !defined(MATLAB_MEX_FILE )
  #include "mingw-std-threads/mingw.future.h"
  #include "mingw-std-threads/mingw.mutex.h"
  #include "mingw-std-threads/mingw.invoke.h"
  #include "mingw-std-threads/mingw.shared_mutex.h"
  #include "mingw-std-threads/mingw.thread.h"
  #include "mingw-std-threads/mingw.condition_variable.h"
#else
  //#include <future>
  #include <mutex>
  //#include <shared_mutex>
  #include <thread>
  #include <condition_variable>
  #include <atomic>
#endif

#include "rang.hxx"
#include "Trace.hxx"
#include "Console.hxx"
#include "Malloc.hxx"
#include "Numbers.hxx"
#include "TicToc.hxx"
#include "ThreadPool.hxx"
#include "Quaternion.hxx"
#include "Table.hxx"
#include "Token.hxx"

namespace Utils {

  #ifndef DOXYGEN_SHOULD_SKIP_THIS
  using std::string;
  #endif

  string basename( char const * const filename );

  template <typename T_int, typename T_real>
  void
  searchInterval(
    T_int                npts,
    T_real const * const X,
    T_real             & x,
    T_int              & lastInterval,
    bool                 closed,
    bool                 can_extend
  );

  #ifndef DOXYGEN_SHOULD_SKIP_THIS
  extern template void searchInterval(
    int32_t             npts,
    float const * const X,
    float             & x,
    int32_t           & lastInterval,
    bool                closed,
    bool                can_extend
  );

  extern template void searchInterval(
    int32_t              npts,
    double const * const X,
    double             & x,
    int32_t            & lastInterval,
    bool                 closed,
    bool                 can_extend
  );

  extern template void searchInterval(
    int64_t             npts,
    float const * const X,
    float             & x,
    int64_t           & lastInterval,
    bool                closed,
    bool                can_extend
  );

  extern template void searchInterval(
    int64_t              npts,
    double const * const X,
    double             & x,
    int64_t            & lastInterval,
    bool                 closed,
    bool                 can_extend
  );
  #endif
}

#endif
```

### Quick search

### Table of Contents

- rang - Colors for your Terminal
- Zstr
- C++ API

«
hide menu

menu
sidebar
»

### Navigation

- index
- toc
- Utils »
- Program Listing for File Utils.hxx

© Copyright 2021, Enrico Bertolazzi.
Created using Sphinx 4.2.0.
